# The Immunopathology of Pulmonary Rejection after Murine Lung Transplantation

**DOI:** 10.3390/cells13030241

**Published:** 2024-01-27

**Authors:** Janne Kaes, Emilie Pollenus, Charlotte Hooft, Hengshuo Liu, Celine Aelbrecht, Seppe Cambier, Xin Jin, Jan Van Slambrouck, Hanne Beeckmans, Pieterjan Kerckhof, Greetje Vande Velde, Dirk Van Raemdonck, Ali Önder Yildirim, Philippe E. Van den Steen, Robin Vos, Laurens J. Ceulemans, Bart M. Vanaudenaerde

**Affiliations:** 1 Laboratory of Respiratory Diseases and Thoracic Surgery (BREATHE), Department of Chronic Diseases and Metabolism, KU Leuven, 3000 Leuven, Belgium; janne.kaes@kuleuven.be (J.K.);; 2 Laboratory of Immunoparasitology, Department of Microbiology, Immunology and Transplantation, Rega Institute for Medical Research, KU Leuven, 3000 Leuven, Belgium; emilie.pollenus@kuleuven.be (E.P.);; 3 Comprehensive Pneumology Center, Institute of Lung Health and Immunity, Helmholtz Munich, Member of the German Center for Lung Research (DZL), 85764 Munich, Germanyoender.yildirim@helmholtz-muenchen.de (A.Ö.Y.); 4 Laboratory of Molecular Immunology, Department of Microbiology, Immunology and Transplantation, Rega Institute for Medical Research, KU Leuven, 3000 Leuven, Belgium; seppe.cambier@kuleuven.be; 5 Biomedical MRI, Department of Imaging and Pathology, KU Leuven, 3000 Leuven, Belgium; 6 Department of Thoracic Surgery, University Hospitals Leuven, 3000 Leuven, Belgium; 7 Department of Respiratory Diseases, University Hospitals Leuven, 3000 Leuven, Belgium

**Keywords:** lung transplantation, pulmonary rejection, chronic lung allograft dysfunction (CLAD), murine orthotopic left lung transplantation, immunology

## Abstract

To improve outcomes following lung transplantation, it is essential to understand the immunological mechanisms that result in chronic graft failure. The associated clinical syndrome is termed chronic lung allograft dysfunction (CLAD), which is known to be induced by alloimmune-dependent (i.e., rejection) and alloimmune-independent factors (e.g., infections, reflux and environmental factors). We aimed to explore the alloimmune-related mechanism, i.e., pulmonary rejection. In this study, we use a murine orthotopic left lung transplant model using isografts and allografts (C57BL/6 or BALB/c as donors to C57BL/6 recipients), with daily immunosuppression (10 mg/kg cyclosporin A and 1.6 mg/kg methylprednisolone). Serial sacrifice was performed at days 1, 7 and 35 post-transplantation (*n* = 6 at each time point for each group). Left transplanted lungs were harvested, a single-cell suspension was made and absolute numbers of immune cells were quantified using multicolor flow cytometry. The rejection process followed the principles of a classic immune response, including innate but mainly adaptive immune cells. At day 7 following transplantation, the numbers of interstitial macrophages, monocytes, dendritic cells, NK cells, NKT cells, CD4+ T cells and CD8+ T and B cells were increased in allografts compared with isografts. Only dendritic cells and CD4+ T cells remained elevated at day 35 in allografts. Our study provides insights into the immunological mechanisms of true pulmonary rejection after murine lung transplantation. These results might be important in further research on diagnostic evaluation and treatment for CLAD.

## 1. Introduction

Lung transplantation is the ultimate treatment for end-stage respiratory failure, but it comprises unique immunological challenges which come with a high risk of adverse effects (e.g., graft failure, solid organ malignancies and side effects of immunosuppression) [1,2]. Long-term outcomes after lung transplantation remain the worst among all solid organ transplants, with a median survival of only six years [3,4]. These low survival rates can be attributed mainly to chronic lung graft dysfunction, which is assumed to be the result of repeated or chronic graft injury that ultimately leads to progressive and pathological fibrosis and eventual graft failure [5]. This clinical phenomenon has long been referred to as ‘chronic rejection’. Basically, transplant rejection can be described as an immunological concept, in which the ‘nonself’ donor organ is recognized by the recipient’s immune system, followed by an immune response including innate and adaptive immune cells [6]. Transplant rejection is historically classified into four stages: hyperacute (mediated by preformed donor-specific antibodies), acute (cellular), antibody-mediated (humoral) and chronic rejection [7].

Initially, obliterative bronchiolitis (OB) was assumed to be the hallmark of chronic pulmonary rejection [8]. Over time, clinical syndromes in lung transplantation were assigned to the immunological concept of chronic rejection, with chronic lung allograft dysfunction (CLAD) as the overarching term [9]. However, CLAD is defined as all late nonspecific and persisting lung function deteriorations after lung transplantation without identifiable cause [3]. This means that CLAD is not a synonym for chronic transplant rejection. Currently, at least three clinical phenotypes are described in CLAD, including ‘bronchiolitis obliterans syndrome’ (BOS), ‘restrictive allograft syndrome’ (RAS) and a mixed phenotype [1].

The exact underlying mechanisms of CLAD are not completely understood; however, this disease is the result of a complex interplay between various multifactorial mechanisms including alloimmune-dependent and -independent processes [10]. Alloimmune factors are associated with transplant rejection, which involves the immune response elicited by the ‘nonself’ donor. By using immunosuppressants, an attempt is made to suppress this inevitable immune response, yet lung transplant recipients often develop rejection episodes. Non-alloimmune-related factors also contribute to CLAD, such as ischemia-reperfusion injury and respiratory infections, which augment the chronic inflammatory milieu persisting in the allograft lung [11].

There are multiple uncontrollable/variable aspects within the follow-up of human lung transplant recipients (e.g., immunosuppressive regimen, genetic variability, infections and other complications). Therefore, it is desirable to study the alloimmune processes in animal models, as this allows us to study the rejection process in a controlled setting [11]. We used a mouse model of orthotopic left lung transplantation to study the alloimmune response [12].

We hypothesize that the immune response preceding pulmonary rejection is an interaction between the different components of the immune system. The objective of this study is to map the sequence of immunological diversity after murine lung transplantation in a sequential manner. It is crucial to improve our insights into the underlying immunological mechanisms of rejection (alloimmune-dependent) to better understand this complex disease and to identify future treatment targets.

## 2. Materials and Methods

### 2.1. Orthotopic Murine Lung Transplant Model

All animals were purchased from Janvier Labs (Le Genest-Saint-Isle, France) and transplanted between eight and ten weeks of age. Recipients were always male C57BL/6N (H-2Kb) and donors were either male C57BL/6N (H-2Kb) for isograft transplantation or male BALB/c (H-2Kd) for allograft transplantation. The experimental procedure was approved by the Ethics Committee for Animal Research at KU Leuven (P194/2019). All mice were housed in a conventional facility with individually ventilated cages (IVC) and received ad libitum standard chow and water.

Orthotopic left lung transplantation was adapted from the technique described by Jungraithmayr et al. [13,14]. Briefly, donor mice were anesthetized with a mixture of xylazine (100 mg/kg; Xyl-M, VMD, Arendonk, Belgium) and ketamine (10 mg/kg; Nimatek, Dechra, Lille, Belgium) administered via an intraperitoneal injection. The chest was opened and 100 μL of heparin (LEO Pharma, Lier, Belgium) was injected into the vena cava inferior. Subsequently, lungs were flushed with 5 mL cold sterile saline through the right ventricle. Pulmonary artery, bronchus and pulmonary vein were dissected and respectively cuffed with 24-, 20- and 22-gauge cuffs. Donor lungs were stored at 4 °C until implantation.

Recipient mice were anesthetized with isoflurane (Iso-Vet, Piramal Critical Care, Voorschoten, The Netherlands) in an induction box, subsequently intubated, connected to the ventilator (UNO microventilator) and placed on a heating pad. The animals were maintained under general anesthesia with a mixture of 2.5% isoflurane and the remaining part half-air and half-oxygen, with a respiratory rate of 120 beats per minute (bpm) and a tidal volume of 300 µL. The left native lung was retracted laterally by placing a curved clamp to expose the hilum. The hilum was dissected to separate the structures from each other. Pulmonary vein and artery were separately closed; afterwards, the cuffed donor structures were inserted in the following order: pulmonary artery, bronchus and pulmonary vein. They were then connected with 10-0 sutures. The native left lung was removed, and the chest was closed with two 6-0 sutures.

After surgery, the mice were allowed to recover on a heating pad overnight and received buprenorphine (Vetergesic, Ecuphar, Breda, The Netherlands) for the following 72 h. Both isografts and allografts received daily immunosuppression subcutaneously consisting of 10 mg/kg/day cyclosporine (Sandimmun^®^, Novartis, Vilvoorde, Belgium) and 1.6 mg/kg/day methylprednisolone (SoluMedrol^®^, Pfizer, Puurs-Sint-Amands, Belgium) starting immediately after transplantation (12 min 25 s ± 3 min 01 s after reperfusion) and continuing until the end of follow-up. The mouse and graft survival was between 80–90% and was equal for isografts and allografts. Graft failures were excluded from further study.

### 2.2. Study Design

**Figure 1:**
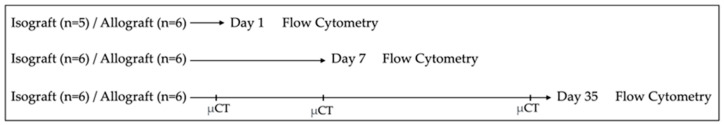
Study design. Orthotopic left lung transplantation was performed to create isografts (C57BL/6 → C57BL/6) and allografts (BALB/c → C57BL/6). Serial sacrifice was performed at day 1, day 7 and day 35 after murine lung transplantation. Following sacrifice, pulmonary cells were isolated and analyzed using multicolor flow cytometry. Longitudinal in vivo microCT was performed at days 1, 7 and 35 in the mice of the group used for flow cytometry sacrificed at day 35. µCT = (in vivo) microCT.

The study design is illustrated in Figure 1. Serial sacrifice was performed at three different time points, at postoperative days 1, 7 and 35 (possibly with one day variation). To determine the immune cell profile, flow cytometry was performed on the complete left lung of allografts and isografts (*n* = 6/group/time point, exception *n* = 5 for isografts at day 1 due to unforeseen logistical circumstances). Longitudinal, nonterminal in vivo microCT imaging was performed on the same allografts and isografts (*n* = 6/group) of the flow cytometry experiment (sacrifice day 35) at days 1, 7 and 35. Terminal exsanguination with blood collection was performed retro-orbitally, under sedation, at time of sacrifice. Cyclosporine A trough levels were measured in 100 µL whole blood and analyzed using a sequential enzyme immunoassay (Dimension^®^ RXL, Siemens Medical solutions, Diamond diagnostics, Holliston, MA, USA). Additional isografts and allografts were transplanted to obtain histology (*n* = 4/group/timepoint).

### 2.3. In Vivo Microcomputed Tomography

Free-breathing mice were scanned with a whole-body small animal microCT scanner (SkyScan 1278, Bruker, Belgium). Animals were anesthetized with isoflurane (2% in pure oxygen) and placed in supine position. The following scan parameters were used: 50 kVp X-ray source, 1 mm aluminum X-ray filter, 346 µA current and 150 ms exposure time per projection, acquiring projections with 0.9° increments over a total angle of 220°. Acquisition resulted in respiratory weighted reconstructed 3D dataset with isotropic voxel size of 51.4 µm in a total scanning time of 3 min, associated with a radiation dose exposure of 69–89 mGy [15]. The images were reconstructed with the following parameters: smoothing of 1, beam-hardening correction of 10%, post-alignment and ring artefact reduction optimally set for each individual scan. Images were processed and calibrated to Hounsfield units (HU) as described previously [16]. Image reconstruction, analysis and quantification were performed using NRecon (V1.7.0.4), DataViewer (V1.5.4.0) and CTAn (V1.16.8.0+) software provided by the manufacturer (Bruker, Belgium). Quantification of total lung volume and mean lung density was performed for a manually delineated region of interest (ROI), resulting in a volume of interest (VOI) on the transversal microCT images at end-expiration.

### 2.4. Isolation of Pulmonary Cells

Pulmonary cells were isolated as previously described [17]. During dissection, lungs were extracted and collected in RPMI buffer (RPMI GlutaMAX (Gibco, Thermofisher Scientific, Dilbeek, Belgium) + 5% FCS + 1% penicillin/streptomycin (Gibco)) with 0.1% β-mercaptoethanol at room temperature. Lungs were minced and incubated for 30 min at 37 °C in digestion medium, consisting of 2 mg/mL collagenase D and 0.1 mg/mL DNase I in RPMI buffer. Subsequently, lung tissue was homogenized using a 20-gauge needle and new digestion medium was added, followed by a second incubation period of 15 min at 37 °C. Cells were washed and red blood cell lysis was performed using 0.83% NH_4_Cl/10 mM Tris at 37 °C and cells were passed through a 70 µm nylon cell strainer. After a last washing step, live leukocytes were counted in a Bürker chamber.

### 2.5. Flow Cytometry of Pulmonary Cells

A total of 1.5–3 million pulmonary cells were used for surface staining. Cells were incubated with a viability dye in combination with Mouse Fc block (MACS Miltenyi Biotec., Leiden, The Netherlands) for 15 min at room temperature. After washing twice, the cells were stained with a panel of monoclonal antibodies (Table S1) dissolved in Brilliant stain buffer (BD Biosciences, Erembodegem, Belgium) for 20 min at 4 °C. After surface staining, 100,000 or 200,000 live single cells were analyzed per sample, depending on the panel, with a BD LSR Fortessa Flow cytometer (BD Biosciences). Two different panels were used for myeloid and lymphoid cells (Table S1). Multicolor flow cytometry was conducted in close collaboration with the laboratory of Immunoparasitology (Rega Institute, KU Leuven). Data were analyzed with FlowJo v10 software (FlowJo LLC, Ashland, OR, USA). Cells were gated according to the gating strategies in the Supplementary figures (Figures S1 and S2) and positive gates were defined using fluorescence minus one (FMO) controls. In order to calculate the absolute numbers of each cell type, the absolute number of CD45+ cells was first calculated by multiplying the percentage of CD45+ cells among the live cells by the total number of cells counted using the Bürker chamber. Subsequently, the absolute numbers of immune cell subsets were calculated by multiplying the percentage of the CD45+ cells by the absolute number of CD45+ cells calculated earlier.

### 2.6. Histology

The lungs were in vivo ventilated and perfused with saline, followed by perfusion with 4% paraformaldehyde (PFA) through the pulmonary artery at time of sacrifice. The trachea was ligated at total lung inflation and lungs were fixed in 4% PFA at 4 °C for 24 h. The grafts were subsequently processed into 4 µm formalin-fixed paraffin-embedded (FFPE) sections. Sections were stained with hematoxylin and eosin (H&E) and Masson’s trichrome.

### 2.7. Statistical Analysis

Data analysis was performed using GraphPad statistical software (Prism, version 9.3.1, San Diego, CA, USA). To compare the different groups, one-way ANOVA was used. To compare the different time points in isografts or allografts, a mixed effects model with Šidák’s multiple comparisons test was conducted. To compare isografts and allografts, a mixed effects model with Tukey’s multiple comparisons post hoc test was performed. A *p*-value of ≤0.05 was considered significant.

## 3. Results

### 3.1. Survival after Murine Orthotopic Single-Lung Transplantation

Orthotopic left lung transplantation was performed with age- and sex-matched donor C57BL/6 (*n* = 5–6/time point) or BALB/c (*n* = 6/time point) mice into C57BL/6 recipient mice (average age 9.35 ± 0.63 weeks) with daily immunosuppression, which resulted respectively in syngeneic (isograft) and major mismatched (allograft) transplanted mice. Eight mice had to be excluded due to surgical failure (8/81; 10%), this was observed macroscopically either as an enlarged, dark red appearance due to pulmonary venous thrombosis or as shrunken yellow lungs. Seven of these eight mice were replaced by new successful transplantations. The first warm ischemic time from donor exsanguination until flush was 2 min 32 s ± 36 s. The total cold ischemic time (start of flush until reperfusion) was 1 h 06 min 38 s ± 10 m 49 s, and the second warm ischemic donor time (start of placement of donor organ inside recipient until reperfusion) was 13 min 27 s ± 3 min 06 s. Overall cyclosporine blood trough levels did not differ significantly between isografts (mean 744.9 ± 213.3 µg/L) and allografts (684.7 ± 238.6 µg/L) (*p* = 0.54). In addition, no significant differences were found within groups; only a significant increase in cyclosporine levels was observed at day 35 compared with day 7 in isografts (*p* = 0.014) (Figure S3).

### 3.2. Alteration in Lung Density after Allograft Transplantation

**Figure 2:**
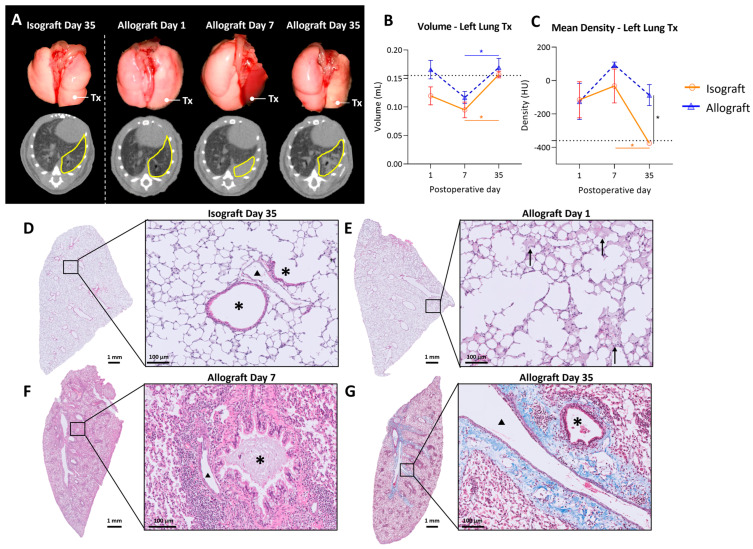
In vivo microCT imaging and histology of the murine orthotopic left lung transplant model. (**A**): Representative macroscopic pictures of lungs with the corresponding in vivo microCT images. The left transplanted lung is indicated with Tx on the pictures and delineated in yellow on microCT sections. (**B**): Evolution of total lung volume of left transplanted lung. Volumes were calculated based on end-expiratory microCT images. The dotted line indicates 0.155 mL, representing a normal left lung volume of C57BL/6 (average of 6 healthy C57BL/6). (**C**): Mean lung density difference in left transplanted lung, calculated from end-expiratory microCT images. The dotted line indicates −360 HU, representing the density of a normal C57BL/6 left lung (average of 6 healthy C57BL/6). (**D**): Isografts (C57BL/6 → C57BL/6), day 35. Asterisks (*) indicate airways and arrowheads (▲) indicate blood vessels. (**E**): Representative H&E image of an allograft (BALB/c → C57BL/6), day 1, interstitial and intra-alveolar edema indicated with arrows. (**F**): H&E image of an allograft, day 7, bronchovascular lymphocytic infiltrates. (**G**): Masson trichrome staining of allograft, day 35, collagen deposition indicated in blue. Data are expressed as mean with SEM. * *p* ≤ 0.05. Tx = transplanted; HU = Hounsfield units.

Macroscopic evaluation showed no major changes in isografts at any time point, while allografts macroscopically demonstrated color fading to yellow with darker patches distributed over the lung 35 days after transplantation (Figure 2A). Transplanted mice were monitored using high-resolution low-dose in vivo microcomputed tomography (microCT) at days 1, 7 and 35 after transplantation. Representative in vivo microCT images are shown in Figure 2A. Visual evaluation of the microCT images showed normal morphology at day 1 in isografts and allografts. However, at day 7 the transplanted allograft lung was completely consolidated on microCT, which corresponded to an atelectatic, non-ventilating lung in vivo. At day 35, a patchy distribution of denser areas in the allografts was observed on microCT. In contrast, isografts presented a normal anatomy of the lungs at days 7 and 35, both macroscopically and on microCT.

Quantification of the lung graft volume revealed no significant differences between isografts and allografts over time. Lung volume increased from day 7 to day 35 post-transplantation in both the isograft (*p* = 0.04) and allograft groups (*p* = 0.008) (Figure 2B). Mean lung density, corresponding to parenchymal attenuation, was significantly higher in allografts (−87.2 ± 154.4 HU) compared with isografts (−375.6 ± 26.9 HU) at postoperative day 35 (*p* = 0.016). Moreover, lung density decreased from day 7 to day 35 in isografts (*p* = 0.043) (Figure 2C). Isografts had a normal preserved parenchymal appearance without cellular infiltrates at any time point (Figure 2D). In allografts, intra-alveolar edema was observed at day 1, showing a multifocal exudate distributed both centrally and peripherally (Figure 2E). At day 7, allografts demonstrated dense mononuclear cellular infiltrates, consisting mainly of lymphocytes or histiocytes (Figure 2F). At day 35, allografts showed abundant foamy macrophages within the alveoli (Figure 2G). Moreover, bronchovascular fibrosis was noted on Masson’s trichrome staining (Figure 2G).

### 3.3. Pulmonary Myeloid Cells after Murine Lung Transplantation

**Figure 3:**
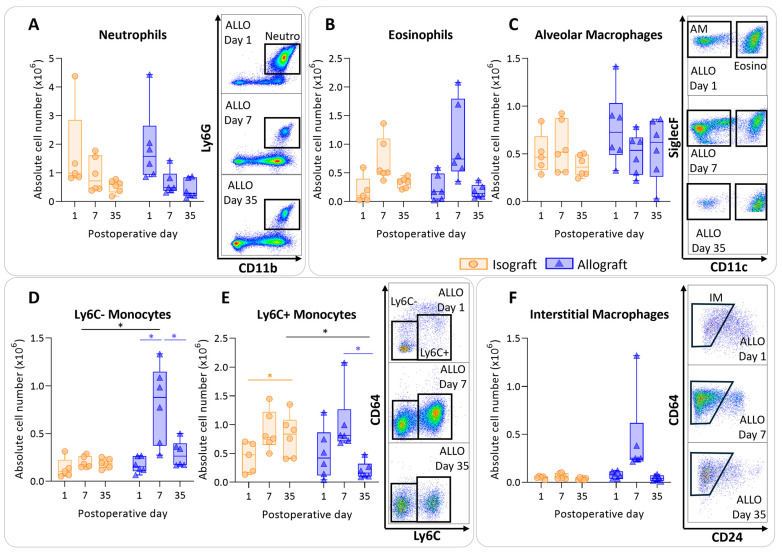
Dynamics of the myeloid cell population in the left transplanted lung after murine orthotopic left lung transplantation. Isograft, depicted in orange, (C57BL/6 → C57BL/6) and allograft, depicted in blue, (BALB/c → C57BL/6) combinations were used at indicated days after transplantation. (**A**): Absolute numbers of innate immune cells including neutrophils (CD45+ CD11b+ Ly6G+). (**B**): Eosinophils (CD45+ CD11b+ SiglecF+ CD11c−). (**C**): Alveolar macrophages (CD45+ SiglecF+ CD11bint CD11c+). (**D**): Ly6C- nonclassical monocytes (CD45+ CD11bhi MHC-II− Ly6C−). (**E**): Ly6C+ inflammatory monocytes (CD45+ CD11bhi MHC-II− Ly6C+). (**F**): Interstitial macrophages (CD45+ CD11bhi MHC-II+ CD64+ CD24−). Data are shown as box-and-whisker plots (box: median with interquartile range, whiskers: full data distribution), with each data point representing an individual mouse sample. * *p* ≤ 0.05. ALLO = allografts; Neutro = neutrophils; Eosino = eosinophils; AM = alveolar macrophages; IM = interstitial macrophages.

To determine the immunological profile of the pulmonary leukocytes in pulmonary rejection after murine orthotopic left lung transplantation, leukocytes were isolated and analyzed using multicolor flow cytometry. Isografts and allografts showed similar patterns concerning granulocytes (neutrophils and eosinophils). The numbers of neutrophils did not significantly differ between and within groups; however, over time neutrophils appeared to decrease in both groups, resulting in a significant time effect (*p* = 0.012) (Figure 3A). Eosinophils showed a similar trend; there were no significant changes between and within groups, but they seemed to be increased in both groups at day 7 (time effect *p* = 0.002) (Figure 3B). The number of alveolar macrophages remained consistent over time, with no significant alterations among groups (Figure 3C). The number of nonclassical, patrolling, Ly6C- monocytes were increased in allografts compared with isografts at day 7 (*p* = 0.04). The number of Ly6C- monocytes was significantly higher at day 7 compared with day 1 and day 35 in allografts (*p* = 0.026; *p* = 0.026) (Figure 3D). Inflammatory, Ly6C+ monocytes were significantly higher at day 7 compared with day 35 in allografts (*p* = 0.03) (Figure 3E). Counts of interstitial macrophages appeared to be higher at day 7 in allografts; however, this was not significant due to an outlier (*p* = 0.23) (Figure 3F).

### 3.4. Antigen-Presenting Cells after Murine Lung Transplantation

**Figure 4:**
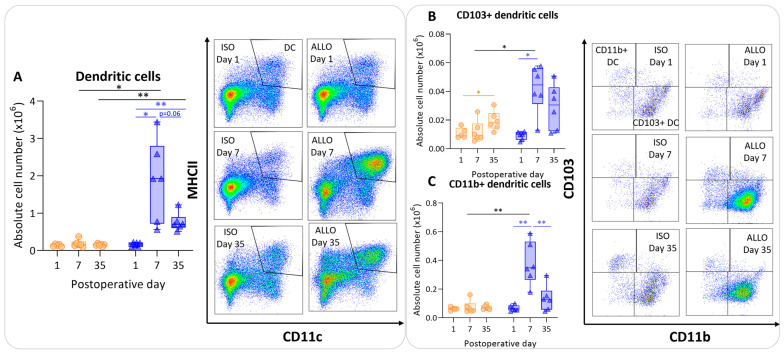
Antigen-presenting cells after murine lung transplantation. Isograft, depicted in orange, (C57BL/6 → C57BL/6) and allograft, depicted in blue, (BALB/c → C57BL/6) combinations were used at indicated days after transplantation. (**A**): Absolute numbers of dendritic cells (CD45+ SiglecF− MHC-II+ CD11c+). (**B**): CD103+ dendritic cells (CD45+ SiglecF− MHC-II+ CD11c+ CD103+ CD11b−). (**C**): CD11b+ dendritic cells (CD45+ SiglecF− MHC-II+ CD11c+ CD103− CD11b+ CD24+ CD64−). Data are shown as box-and-whisker plots (box: median with interquartile range, whiskers: full data distribution), with each data point representing an individual mouse sample. * *p* ≤ 0.05; ** *p* ≤ 0.01.

The number of dendritic cells was significantly higher at day 7 compared with day 1 in allografts, and appeared to be lower at day 35, although non-significantly (*p* = 0.06). However, dendritic cell counts remained significantly higher at day 35 compared with day 1 within allografts (*p* = 0.003) (Figure 4A). The isografts’ dendritic cells remained stable over time. The dendritic cell count was higher in the allografts compared with the isografts at day 7 and day 35 (*p* = 0.04; *p* = 0.004) (Figure 4A). CD103+ migratory dendritic cells were higher at day 7 (*p* = 0.015) in allografts versus isografts (Figure 4B). Similarly, CD11b+ dendritic cell numbers were higher in allografts compared with isografts at day 7 (*p* = 0.01); these are identified as lymphoid tissue-resident classic dendritic cells (Figure 4C).

### 3.5. Pulmonary Lymphoid Cells after Murine Lung Transplantation

**Figure 5:**
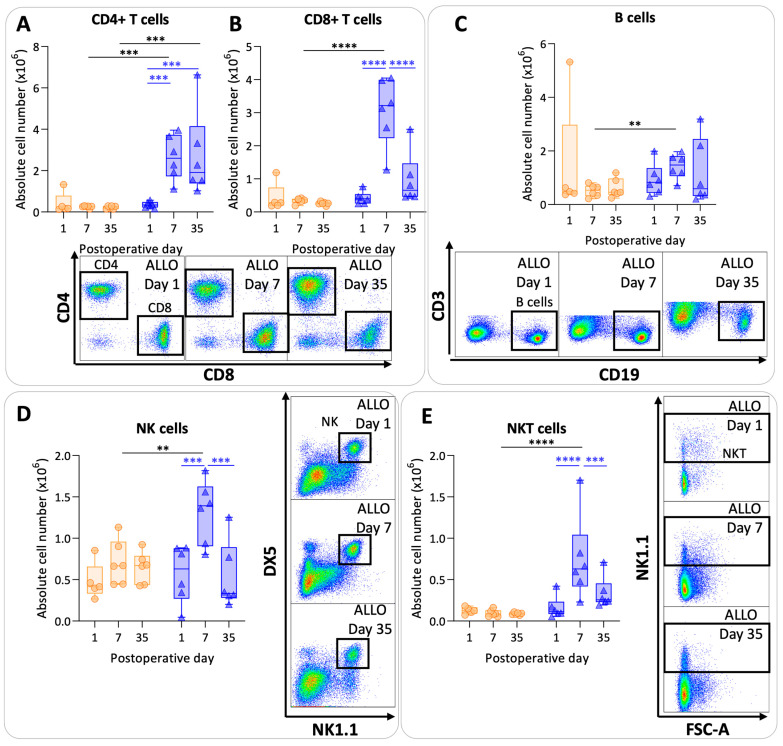
Lymphoid pulmonary cells after murine lung transplantation. Isograft, depicted in orange, (C57BL/6 → C57BL/6) and allograft, depicted in blue, (BALB/c → C57BL/6) combinations were used at indicated days after transplantation. (**A**): Absolute numbers of adaptive immune cells including CD4+ T cells (CD45+ CD3+ NK1.1− CD4+). (**B**): CD8+ T cells (CD45+ CD3+ NK1.1− CD8+). (**C**): B cells (CD45+ CD3− NK1.1− B220+). (**D**): NK cells (CD45+ CD3− NK1.1+ DX5+). (**E**): NKT cells (CD45+ CD3+ NK1.1+). Data are shown as box-and-whisker plots (box: median with interquartile range, whiskers: full data distribution), with each data point representing an individual mouse sample. ** *p* ≤ 0.01; *** *p* ≤ 0.001; **** *p* < 0.0001. NK = natural killer.

CD4+ T cell values were significantly higher at day 7 and day 35 in the allografts compared with the isografts (*p* = 0.0008; *p* = 0.0004) (Figure 5A). In allografts, CD4+ T cell counts increased at day 7 and at day 35 compared with day 1 (*p* = 0.0009; *p* = 0.0007). Numbers of CD8+ T cells were higher at day 7 in allografts compared with isografts (*p* < 0.0001). However, CD8+ T cell values were lower again at day 35 in allografts compared with day 7 allografts (*p* < 0.0001) (Figure 5B). B cells showed variable results with a significant difference between allografts and isografts at day 7 (*p* = 0.01) (Figure 5C). NK cell and NKT cell numbers were significantly elevated at day 7 in the allografts compared with the isografts (*p* = 0.007; *p* < 0.0001) (Figure 5D,E). NK and NKT cell numbers returned back to baseline values at day 35 in allografts compared with day 7 (*p* = 0.0002; *p* = 0.0007).

## 4. Discussion

In this explorative study of pulmonary rejection following murine orthotopic left lung transplantation, we demonstrated a complex interplay of innate and adaptive immune responses, shedding light on the immunological dynamics associated with rejection. Following transplantation, an innate immune response is started in both iso- and allografts. In allografts, this is followed by the infiltration of the main antigen-presenting cells, i.e., dendritic cells. Dendritic cells present antigens to activate the adaptive immune system, including CD4+ T cells, CD8+ T cells and B cells. Day 7 after murine lung transplantation demonstrated a peak in most of the measured immune cells. While the majority of the cells returned back to isograft values, CD4+ T cells remained high at day 35 in allografts. Dendritic cells decreased but were still elevated at day 35 compared with isograft numbers. A similar trend was observed for CD8+ T cells, although it was not significant.

Innate immunity is the first responder after injury. Our results showed an increase in neutrophils at day 1 after transplantation in both iso- and allografts. This can be attributed to the fact that lung transplantation is followed by ischemia-reperfusion injury (IRI). IRI is characterized by an innate immune response including infiltration of host neutrophils into the grafts [18]. In addition, BAL neutrophilia was previously correlated with the development of CLAD [19] and turned into a separate phenotype called ‘azithromycin response allograft dysfunction’ (ARAD). By definition, ARAD is reversible after three to six months of treatment with azithromycin [20]. The reversibility of this phenotype and the absence of neutrophils in the long term in our results might suggest a nuanced role for neutrophils, potentially linked to primary graft dysfunction (PGD) [21,22]. In this way, neutrophils might be indirectly associated with CLAD.

Eosinophils, recognized for their toxic granules and innate immune function, exhibited a transient elevation at day 7 in both iso- and allografts. Despite our result, Verleden et al. demonstrated the association between BAL eosinophilia and CLAD; however, the number of infections was higher in the BAL eosinophilia group compared with the control group [23]. This prompted speculation that eosinophils might be more associated with inflammation resulting from infections, a known risk factor for CLAD, rather than direct contributors to alloimmune rejection, but this deserves more in-depth investigation [24].

Dendritic cells form the bridge between innate and adaptive immunity, and emerged as pivotal players in the rejection process in our mouse model. Their sustained elevation at day 35 in allografts underscores their integral role in the development of rejection, aligning with findings by Vandermeulen et al. that identified increased dendritic cells in CLAD-related phenotypes [25]. By presenting allo-antigens to the recipient’s immune system, a chronic immune response develops; therefore, dendritic cells probably play a crucial role in pulmonary rejection and CLAD [26].

Monocytes, interstitial macrophages, NK cells and NKT cells were increased in the allografts at day 7, probably representing a more acute phase of pulmonary rejection. The role of monocytes and macrophages in lung transplantation remains underexplored, and conflicting results are reported [25,27,28]. NK cells, known for their potent effector functions, may contribute to graft injury. However, NK cells could also mediate allograft tolerance by direct cytotoxicity of donor antigen-presenting cells, as NK cells sense the missing self, presenting a possible dual role in the rejection process [29].

Our findings demonstrated a strong adaptive immune response, primarily dominated by CD4+ and CD8+ T cells, even though the recipient mice received daily cyclosporine. Cell-mediated immunity is a well-known mechanism leading to pulmonary rejection [30,31,32]. B cells displayed variable results in our study, which might be attributed to the relatively short duration of the experiment. Nevertheless, there is growing evidence of the significant role of humoral immunity in CLAD. In multiple mechanistic studies using the mouse transplant model, B cells have often been suggested as one of the key drivers of rejection [33,34,35]. Limited data showed that B cells and immunoglobulins in CLAD patients are strongly present [25,36,37].

According to our data, the pulmonary rejection process adheres to the fundamental principles of a classic immune response, including cellular innate and adaptive elements [38]. This challenges the traditional classification of transplant rejection entities (i.e., acute rejection, antibody-mediated rejection and chronic rejection). The observed peaks in immune cell activation at day 7, followed by a decline into a less active, organized immune response at day 35, suggest a dynamic spectrum of alloimmune responses. Speculatively, lymphocyte exhaustion or replacement of donor cells with recipient cells might contribute to less allogeneic immune cell triggering over time [39].

This study has some limitations, such as the species-specific differences between mice and humans, and the lack of non-transplanted controls. We did not analyze different locations in parallel (e.g., draining lymph nodes, peripheral blood, BAL), as these may have added values for translation toward the clinical setting. Our focus was on evaluating the essential principles of rejection within the transplanted lung and on yielding mechanistic insights into pulmonary rejection, and not yet on providing any new biomarkers for CLAD [40]. However, we explored the immune cells in the spleen (Figure S4) and observed a similar pattern for the immune cells, except that the adaptive immune cells (and most remarkably CD4+ T cells) did not persist to day 35. We considered the variability of the animal model in both the progression and severity of rejection. The variation somewhat resembles the human transplant setting; the variability has multiple causes that are often not known precisely (e.g., surgery-related or interindividual differences). The use of immunosuppression was implemented in this model to simulate the clinical human situation. Despite immunosuppressive therapy, patients and mice still develop pulmonary rejection. It is essential to acknowledge that we have analyzed only the predominant cells of the immune system to construct a comprehensive understanding of rejection on a global scale. However, it is imperative to recognize that the immune system encompasses a considerable degree of diversity and intricacy involving various cell subtypes and interactions. The elucidation provided thus far merely scratches the surface of this complex landscape.

## 5. Conclusions

In conclusion, we described the process of pulmonary rejection in mice. We demonstrated that a classical alloimmune response occurs including both the innate and adaptive immune system. The involvement of nearly all types of immune cells, rather than one specific cell type, poses a challenge for targeted therapeutic strategies [28]. CLAD is by concept defined as a result of both alloimmune-related and alloimmune-unrelated mechanisms. In this study, we have focused solely on the alloimmune-related mechanisms. (Chronic) rejection may be just a small fraction of CLAD, alongside injury driven by non-alloimmune factors, such as infections, reflux and environmental factors like pollution and smoking [41]. The latter directly cause damage to the epithelium and/or endothelium, contributing to chronic inflammation and thereby facilitating allorecognition [42].

## Data Availability

The original contributions presented in the study are included in the article/supplementary material, further inquiries can be directed to the corresponding author.

## Supplementary Materials

The following supporting information can be downloaded at: https://www.mdpi.com/article/10.3390/cells13030241/s1, Figure S1: Gating strategy for myeloid panel for pulmonary leukocytes; Figure S2: Gating strategy for lymphoid panel for pulmonary leukocytes; Figure S3: Cyclosporine A values after murine lung transplantation; Table S1: Markers with associated clone and fluorochrome for the panels used for flow cytometry; Figure S4: Myeloid and lymphoid splenic cells after murine lung transplantation.
