# Supplementary material for: The Immunopathology of Pulmonary Rejection after Murine Lung Transplantation
Source: Cells. 2024 Jan 27;13(3):241. doi: 10.3390/cells13030241 (PMC10854916; doi:10.3390/cells13030241)
Supplement: Supplementary file 1 [file cells-13-00241-s001.zip › cells-2820396-supplementary.pdf]

Supplementary Figures

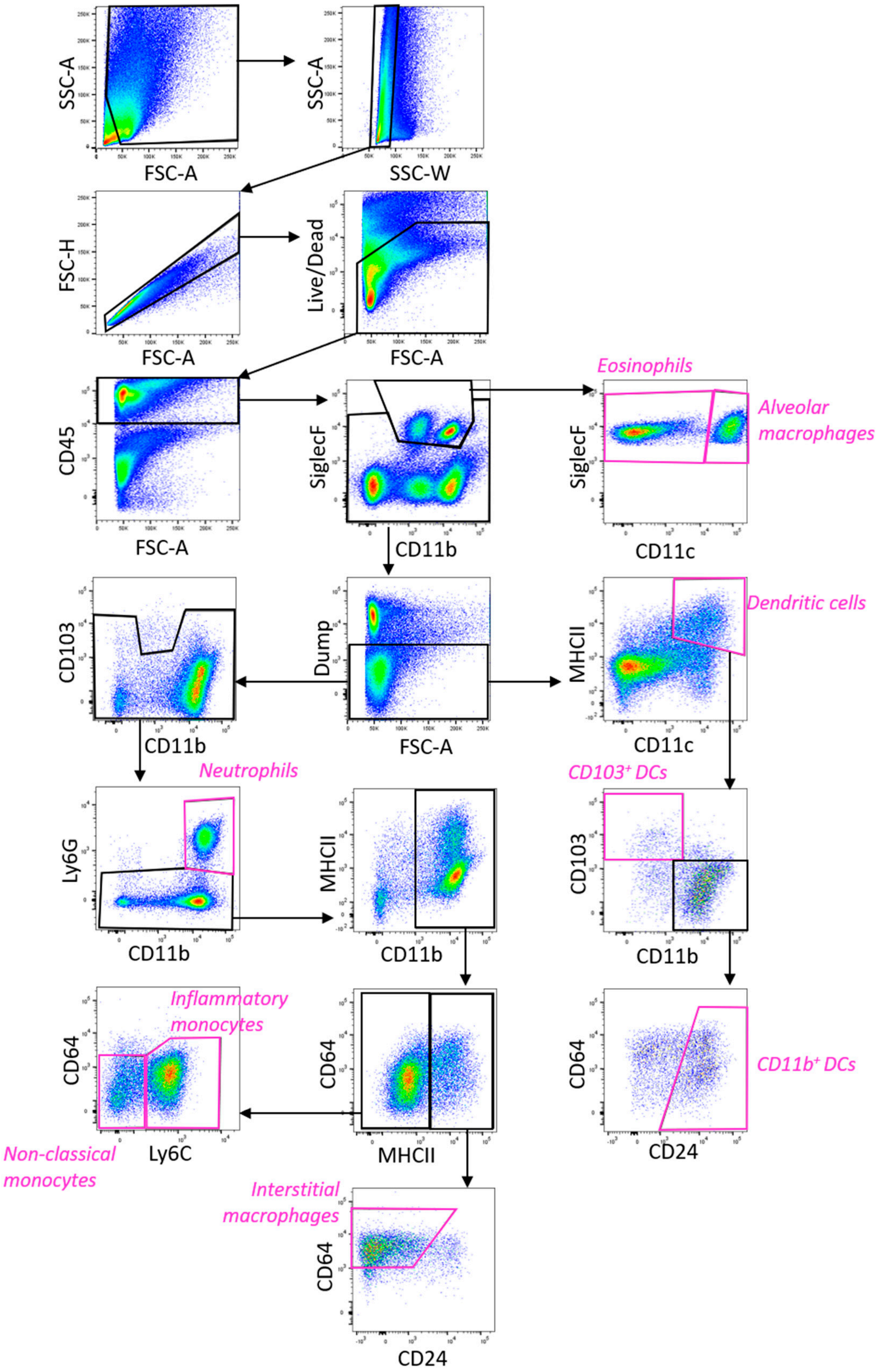

Figure S1: Gating strategy for myeloid panel for pulmonary leukocytes. Lung cells were isolated and stained for flow cytometry. After exclusion of red blood cells, debris and doublets of cells, all live cells (Live/dead) were gated. The leukocytes were identified by CD45 staining. Dump gate includes CD3, CD19, NK1.1.

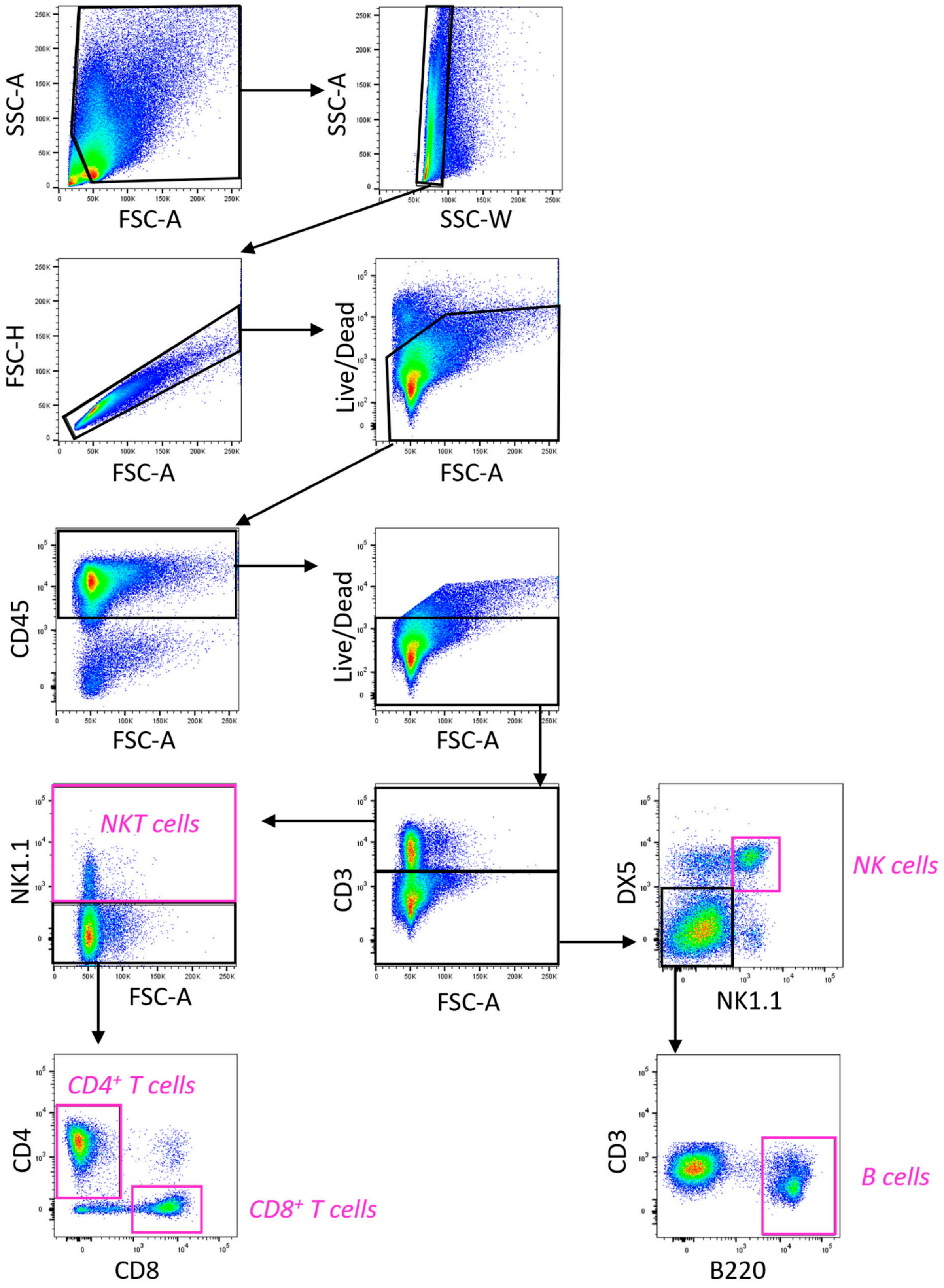

Figure S2: Gating strategy for lymphoid panel for pulmonary leukocytes. Lung cells were isolated and stained for flow cytometry. After exclusion of red blood cells, debris and doublets of cells, all live cells (Live/Dead) were gated.

The leukocytes were identified by CD45 staining. The alveolar macrophages were excluded by plotting Live/Dead versus FSC-A as these cells are autofluorescent and result in false positive staining for lymphoid cell markers.

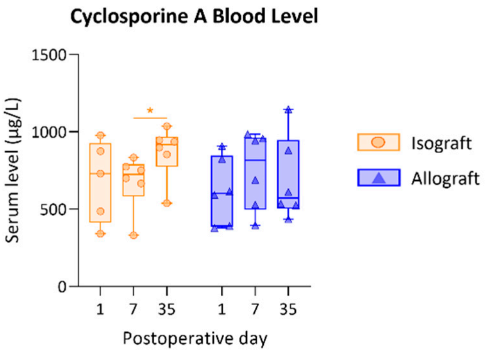

Figure S3: Cyclosporine A values after murine lung transplantation. Orthotopic left lung transplantation was performed to create isografts (C57BL/6 ➝ C57BL/6) and allografts (BALB/c ➝ C57BL/6). Cyclosporine A blood trough levels were measured by sequential enzyme immunoassay in 100 µL whole blood. Data are shown as box-and-whisker plots (box: median with interquartile range, whiskers: full data distribution), with each data point representing an individual mouse sample.

Table S1: Markers with associated clone and fluorochrome for the panels used for flow cytometry.

Panel	Marker	Clone	Fluorochrome	Laser
Myeloid cells	CD45	30-F11	FITC	BLUE
	CD103	2-E7	PerCP-eFluor 710	BLUE
	CD11c	N418	PE-Cy7	YG
	CD64	X54-5/7.1	PE	YG
	CD24	M1/69	PE-CF594	YG
	Siglec F	1RNM44N	eFLUOR 660	RED
	Ly6G	1A8	Alexa fluor 700	RED
	Ly6C	AL-21	APC-Cy7	RED
	CD11b	M1/70	eFLUOR 450	VIOLET
	MHCII	M5/114.15.2	Horizon v500	VIOLET
	CD3	17A2	BV650	VIOLET
	CD19	6D5	BV650	VIOLET
	NK1.1	PK136	BV650	VIOLET
	Live/dead		Zombie UV	UV
Lymphoid cells	CD3	145-2C11	FITC	BLUE
	CD8	53-6.7	PerCP-Cy5.5	BLUE
	NK1.1	PK136	Pe-Cy7	YG
	CXCR3	CXCR3-173	PE	YG
	CD4	RM4-5	APC e780	RED
	DX5	DX5	APC	RED
	B220	RA3-6B2	BV786	VIOL
	CD45	30-F11	BUV395	UV
	Live/dead		Zombie Aqua	VIOL

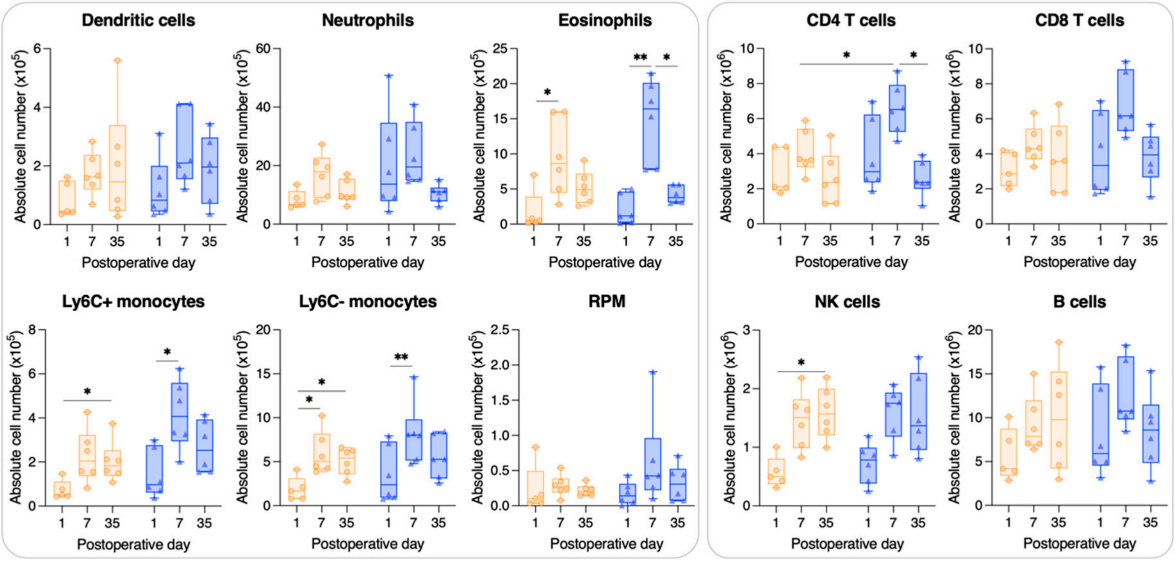

Figure S4: Myeloid and lymphoid splenic cells after murine lung transplantation. Isograft, depicted in orange, (C57BL/6 ➝ C57BL/6) and allograft, depicted in blue, (BALB/c ➝ C57BL/6) combinations were used at indicated days after transplantation. Left panel shows the absolute numbers of splenic, myeloid immune cells including dendritic cells (CD45+ MHCII+ CD11c+); neutrophils (CD45+ CD11b+ Ly6G+); eosinophils (CD45+ SiglecF+ CD11c-); Ly6C+ monocytes (CD45+ CD11b+ MHCII- Ly6C+); Ly6C- monocytes (CD45+ CD11b+ MHCII- LyC6-); RPM (CD45+ MHCII- SiglecF- F4/80+ CD11b-). Right panel includes the absolute numbers of splenic, lymphoid immune cells including: CD4+ T cells (CD45+ CD3+ NK1.1− CD4+); CD8+ T cells (CD45+ CD3+ NK1.1− CD8+); NK cells (CD45+ CD3− NK1.1+ DX5+); B cells (CD45+ CD3− NK1.1− B220+). Data are shown as box-and-whisker plots (box: median with interquartile range, whiskers: full data distribution), with each data point representing an individual mouse sample. * $p \leq 0.05$; ** $p \leq 0.01$. RPM = red pulp macrophages.
